# The Nuclear Receptor and Clock Repressor Rev-erbα Suppresses Myogenesis

**DOI:** 10.1038/s41598-019-41059-7

**Published:** 2019-03-14

**Authors:** Somik Chatterjee, Hongshan Yin, Weini Li, Jeongkyung Lee, Vijay K. Yechoor, Ke Ma

**Affiliations:** 1 Center for Diabetes Research, Department of Medicine, Houston Methodist Research Institute, Houston, TX 77030 USA; 2 Department of Cardiovascular Medicine, Third Affiliated Hospital, Hebei Medical University, Shijiazhuang, 050051 Hebei, China; 3 Department of Diabetes Complications & Metabolism, Beckman Research Institute of City of Hope, Duarte, CA 91010 USA; 4 Diabetes and Beta Cell Biology Center, Division of Endocrinology, Diabetes & Metabolism, Department of Medicine, University of Pittsburgh, Pittsburgh, PA 15213 USA

**Keywords:** Cell biology, Differentiation

## Abstract

*Rev-erbα* is a ligand-dependent nuclear receptor and a key repressor of the molecular clock transcription network. Accumulating evidence indicate that the circadian clock machinery governs diverse biological processes in skeletal muscle, including muscle growth, repair and mass maintenance. The physiological function of Rev-erbα in myogenic regulation remains largely unknown. Here we show that *Rev-erbα* exerts cell-autonomous inhibitory effects on proliferation and differentiation of myogenic precursor cells, and these actions concertedly inhibit muscle regeneration *in vivo*. Mechanistic studies reveal *Rev-erbα* direct transcriptional control of two major myogenic mechanisms, proliferative pathway and the Wnt signaling cascade. Consistent with this finding, primary myoblasts lacking Rev-erbα display significantly enhanced proliferative growth and myogenic progression. Furthermore, pharmacological activation of Rev-erbα activity attenuates, whereas its inhibition by an antagonist promotes these processes. Notably, upon muscle injury, the loss-of-function of *Rev-erbα in vivo* augmented satellite cell proliferative expansion and regenerative progression during regeneration. Collectively, our study identifies Rev-erbα as a novel inhibitory regulator of myogenic progenitor cell properties that suppresses postnatal myogenesis. Pharmacological interventions to dampen Rev-erbα activity may have potential utilities to enhance regenerative capacity in muscle diseases.

## Introduction

*Rev-erbα* is a member of the nuclear receptor superfamily (Subfamily 1 group D member 1, NR1D1), and a key circadian clock repressor that inhibits core clock activator Brain and Muscle Arnt-line 1 (*Bmal1*) transcription^1^. Rev-erbα is highly expressed in metabolic tissues, with known functions in conferring circadian clock integration to glucose, lipoprotein and bile acid metabolism^2,3^. Interestingly, although the Rev-erbα metabolic functions have been extensively studied, its role in skeletal muscle growth and repair processes is largely unknown.

Accumulating evidence indicate that the circadian clock is critical for maintenance of skeletal muscle mass and growth^4^. The circadian clock circuit, composed of an inter-locking molecular network of transcriptional and translational regulators^5,6^, participates in diverse aspects of skeletal muscle functions ranging from oxidative metabolism, sarcomeric structure, contractile performance to muscle mass maintenance^7–11^. A remarkable ~17% of all genes examined in skeletal muscle exhibit circadian oscillations^12^, with many involved in proliferative or growth processes.

Bmal1 is the essential transcription regulator of the clock transcriptional feedback circuit. Bmal1 dimerization with Circadian Locomotor Output Cycles Kaput (CLOCK) activates downstream target genes, *Period* (*Per1*, 2 and 3) and *Cryptochrome* genes (*Cry1* and 2) that in turn inhibits Bmal1/CLOCK activity^5^. *Rev-erbα* potently suppresses *Bmal1* transcription through a shared response element with retinoid acid receptor-related orphan receptor alpha (RORα), the RevRE/RORE^1^. Rev-erbα represses, whereas RORα activates *Bmal1* gene transcription, and this antagonistic regulation elicit *Bmal1* rhythmic oscillation. Interestingly, Rev-erbα itself is a direct target of *Bmal1*, and the *Rev-erbα-Bmal1* regulation constitutes a re-enforcing branch that enhances the robustness of the core clock machinery^1,13^.

Regenerative myogenesis is a highly orchestrated progression involving myogenic precursor cell (MPC) proliferation, differentiation and fusion to form multinucleated myofibers for muscle repair^14,15^. Recent studies indicate that the circadian clock regulation is critical for muscle growth and function^4^. Loss of *Bmal1* leads to severe aging-associated sarcopenia^9^, and *Bmal1* and *CLOCK* are required for myofilament integrity^7^. We recently demonstrated that *Bmal1* promotes myogenic precursor proliferation and differentiation required for skeletal muscle regenerative myogenesis^16,17^, with its ablation leading to significantly impaired satellite cell proliferative expansion and muscle regeneration following injury. Our findings of Bmal1 modulation of MPC properties implicate potential intrinsic clock control in muscle repair that may require additional clock components. Recent cistrome analysis of *Rev-erbα* demonstrate an extensive ~28% overlap with *Bmal1*^18,19^, suggesting that they may exert opposing transcriptional controls of shared targets.

As a ligand-dependent nuclear receptor, *Rev-erbα* synthetic ligands are currently available to interrogate its biological functions and potential pharmacological interventions of the circadian clock^20–22^. Based on *Bmal1* regulation of the myogenic cascade, we hypothesize that the transcription repressor Rev-erbα may inhibit myogenesis to suppress regenerative repair. In the current study, we employed genetic and pharmacological approaches to probe the physiological functions of Rev-erbα in myogenic regulations and muscle regeneration.

## Materials and Methods

### Animals

Mice were maintained in the Baylor College of Medicine vivarium under a constant 12:12 light dark cycle. All animal procedures were conducted in accordance with the Guidelines for the Care and Use of Laboratory Animals and were approved by the IACUC committee of Baylor College of Medicine. Global gene-targeted *Rev-erbα-null (Rev*^*−/−*^*) mice* were generated by the Genetically Engineered Mouse Core at Baylor College of Medicine using targeted embryonic stem cells (Velocigene ES clone 11705A-E7, KOMP), as described previously^23^. Male mice of 8–10 weeks of age were used for the study. Homozygote *Rev*^*−/−*^ mice were obtained through heterozygote breeding, with age-matched littermate WT mice as controls.

### Primary myoblast isolation and culture

Primary myoblasts were isolated from hind limb muscle of 4 week-old mice, as described^16^. Briefly, muscles were minced and subjected to collagenase digestion and seeded on collagen-coated plates, using pre-plating to deplete fibroblasts. Cells were subsequently expanded in myoblast growth media for 6 passages. Purity of myoblasts obtained was confirmed by uniform differentiation into myosin heavy chain (MyHC)-positive myotubes. Proliferative myoblast cultures were maintained in F-10 medium supplemented with 20% FBS and 5 ng/ml bFGF. 2% horse serum supplemented DMEM was used for induction of differentiation. Crushed muscle extract (CME) was obtained as described previously^17^, from leg muscles from 8–10 week old C57BL/6 mice gently pressed using blunt forceps. Supernatant after 2 hours 4 °C incubation in Tris-buffered saline was obtained and protein concentration determined. Primary myoblast were treated by muscle extract at a protein concentration of 300 μg/ml.

### Microarray Analysis

Mouse WG-6 V2.0 whole genome expression arrays were obtained from Illumina. Total RNA from three independent primary myoblast samples were used to synthesize cRNA using Illumina TotalPrep RNA amplification kit (Thermo Scientific). Biotin Labeling and hybridization of cRNA were performed according to manufacturer’s protocol, as described^24^. Arrays were scanned using BeadArray Reader and the data extracted using Illumina BeadStudio software. Unmodified microarray data were background-subtracted and quantile-normalized using Lumi and analyzed with the limma package within R to test differentially gene expression^25^. All analyses were corrected for multiple hypothesis testing^26^, and effects determined as significant with an adjusted *p*-value ≤ 0.05. The microarray dataset was deposited in NCBI Gene Expression Omnibus accession number GSE110765. KEGG pathway analysis was carried out among significantly regulated genes to identify enriched biological processes.

### EdU labelling and proliferation assay *in vitro*

Primary myoblasts seeded on collagen-coated chamber slides were labeled with 10 μM 5-ethynyl-2′-deoxyuridine (EdU) for 6 hours. EdU incorporation was detected by Click-iT Imaging Kit (Invitrogen) with Alexa 488 Fluorophore and DAPI was used to label nuclei. Total number of EdU^+^ cells was calculated from 4 representative fields and proliferation rate calculated as percentage of EdU^+^ to total nuclei.

### Quantitative TUNEL assay

Primary myoblasts were plated on collagen-coated 96 well plate. Cells were treated with endonuclease to induce apoptosis as positive control. Terminal deoxynucleotidyl transferase *in situ* labeling and detection of apoptosis was performed 24 hours after seeding by colorimetric TACS apoptosis detection kit (Trevigen), according to manufacturer’s protocol.

### Analysis of Wnt signaling activity

Wnt activity in primary myoblasts, as assessed by β-catenin intracellular localization at basal state without stimulation and after Wnt3a stimulation, was carried out as described^16^. Briefly, primary myoblasts were treated by unconditioned or conditioned media from L3-Wnt3a (40%) for 10 minutes, and nuclear fraction was collected by ultracentrifugation. β-catenin level in the nuclear and total cellular extracts were analyzed by immunoblot. Ratio of the nuclear fraction to total β-catenin was used to assess Wnt signaling activity after normalization to TBP. TopFlash Wnt luciferase reporter assay was performed as described^16^. SR9011 and SR8278 (10 nM) were added for 8 hours prior to Wnt3a stimulation overnight. Luciferase activity was measured using Dual-Glo luciferase assay (Promega) 24 hours after treatment and values normalized to Renilla readings.

### Muscle Injury Model

Cardiotoxin (CTX, 30 μl of 10 μM solution) was injected into Tibialis Anterior muscle along the longitudinal axis, as described^27,28^, with PBS-injected animals as controls. Muscles on post-injury days were collected at 4 PM, the time of CTX injection, to ensure comparable circadian time sampling. Tissues were fixed in 10% paraformaldehyde for paraffin sections, or frozen in liquid nitrogen-cooled isopentane for cryosections. Histological analysis was performed in middle region of the Tibialis Anterior. Myofiber cross-section areas were measured in H/E staining sections of five representative fields of (150 myofibers per mice), using the Nikon NIS-Elements software. For EdU *in vivo* labeling, mice were injected i.p. of EdU (5 mg/Kg) 6 hours prior to sample collection. Click-iT EdU kit (ThemoFisher) was used for EdU detection.

### Histology, immunofluorescence staining and morphometric analysis

Muscles were snap frozen in liquid-nitrogen cooled isopentane and embedded in OCT for cryosections. 10 μm cryosections of middle region of the injured TA muscle were used for immunostaining. Sections were fixed in 4% paraformaldehyde and permeabilized by 0.2% Triton X-100, and endogenous IgG blocked prior to primary antibody incubation. The number of Pax7^+^ satellite cells or EdU^+^Pax7^+^ proliferative satellite cells was determined from three representative fields of each mice, and percentage of Pax7^+^ satellite cells to total nuclei or myofibers were calculated.

### Immunoblot analysis

20–40 µg of total protein was resolved on SDS-PAGE gel followed by immunoblotting after nitrocellulose membrane transfer, as described previously (Chatterjee *et al*., 2013), and developed by chemiluminescence (Supersignal; Pierce Biotechnology). Source and dilution information of primary antibodies are included as Suppl. Table 1. Specific secondary antibodies were used at a dilution of 1:3000.

### RNA extraction and quantitative reverse-transcriptase PCR analysis

RNeasy miniprep kits (Qiagen) were used to isolate total RNA from snap-frozen muscle tissues and cells, respectively. cDNA was generated using q-Script cDNA Supermix kit (Quanta Biosciences) and quantitative PCR was performed using a Roche 480 Light Cycler with Perfecta SYBR Green Supermix (Quanta Biosciences). Relative expression levels were determined using the comparative Ct method to normalize target genes to 36B4 internal control. Primer sequences used are listed in Suppl. Table 2.

### Chromatin Immunoprecipipation (ChIP)-qPCR Analysis

Immunoprecipitation was performed using a Rev-erbα antibody specific for IP (Proteintech 14506-1-AP), or control rabbit IgG with Magnetic Protein A/G beads, using the Magna ChIP A/G kit (Millipore), similarly as described previously^29,30^. Briefly, C2C12 myoblasts were fixed using 1% formaldehyde for 10 minutes, lysed and sonicated using Biodisruptor to shear the chromatin. The immunoprecipitated chromatin fragments were eluted, treated with proteinase K and purified using DNA purification column. Real-time PCR using Perfecta SYBR Green Supermix (Quanta Biosciences) was carried out using 1 µl of eluted chromatin for each reaction in triplicates with specific primers for predicted RORE response elements identified on the target promoters or first intron. Known Rev-erbα binding site in Bmal1 first intron was included as positive control and TBP first exon genomic primers as negative control. ChIP rimer sequences used are listed in Supplementary Material Table S3. Values were expressed as fold enrichment normalized to IgG control.

### Statistical analysis

Data are presented as mean ± SE. One way ANOVA or two-tailed Student’s *t*-test were performed as appropriate as indicated. Non-parametric Kruskal–Wallis ANOVA for entire dataset and Mann-Whitney test for individual cross section area categories were used to assess statistical difference in myofiber size distribution. Statistical analyses were carried out using Prism by GraphPad. P < 0.05 was considered statistically significant.

## Results

### Rev-erbα is highly enriched in skeletal muscle and responds to myogenic stimuli

**Figure 1:**
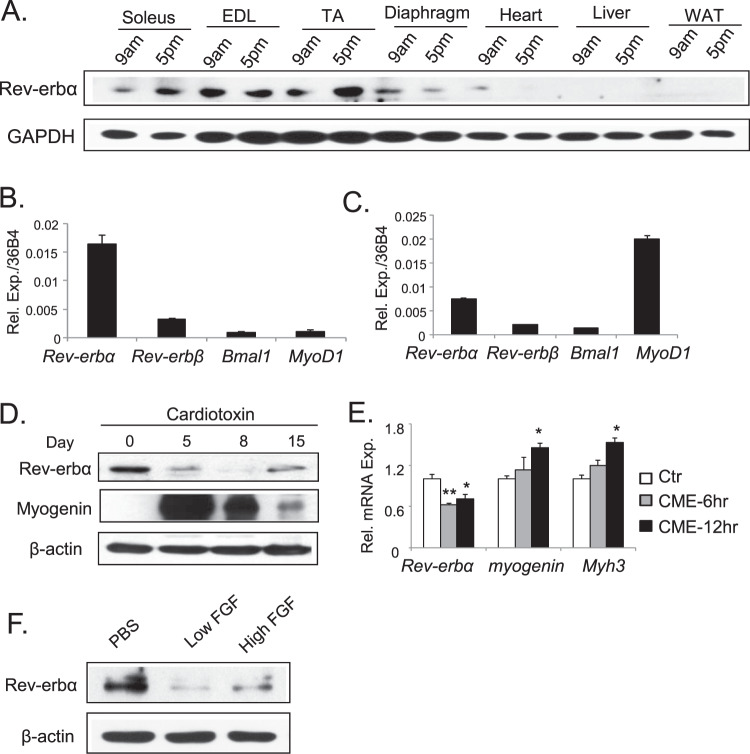
Rev-erbα expression and dynamic regulation in skeletal muscle and myoblasts. (**A**) Immunoblot analysis of time-dependent expression profile of Rev-erbα protein in distinct tissues at 9 AM and 5 PM. Each lane represents pooled protein sample of n = 5. (**B**,**C**) RT-qPCR analysis of comparison of *Rev-erbα* and related core circadian clock gene expression in mice quadricep (**B**, n = 6), and C2C12 myoblasts (**C**, n = 3). (**D**) Immunoblot analysis of dynamic expression profiles of Rev-erbα and myogenin during 15 days post cardiotoxin-induced injury of Tibialis Anteria (TA) muscle (n = 5–6/time point). (**E**) RT-qPCR analysis of *Rev-erbα* and myogenic gene expression in primary myoblasts (n = 4/group) following treatment of crushed muscle extract (CME) at a protein concentration of 300 μg/ml, for 6 and 12 hours respectively. ***P ≤ 0.05 or 0.01 by Student’s t test. (**F**) Suppression of Rev-erbα protein expression as analyzed by immunoblot analysis in response to myogenic stimuli Fgf2 treatment in primary myoblasts for 24 hours (n = 4/group). Fgf2 concentration: low: 2.5 ng/ml, high: 25 ng/ml.

To study the function of Rev-erbα in skeletal muscle, we examined the abundance of its protein expression among distinct tissues and muscle fiber types at 9 AM and 5 PM. Interestingly, at both times of the day, Rev-erbα protein is more abundant in skeletal muscles as compared to heart, liver and white adipose tissue (Fig. 1A). Among the muscle types, a distinct diurnal pattern of expression with higher expression at 5 PM than 9 AM is observed in slow-twitch Type I soleus and mixed fiber type of Tibialis Anteria, but not evident in the fast-twitch extensor digitalis longus (EDL). The average expression in diaphragm is low as compared to other muscles. Thus the current study, we confined our sample collection to 4–5 PM when Rev-erbα protein, particularly in Tibialis Anterior, is more abundant. Further analysis of clock components in Tibialis Anterior reveal that *Rev-erbα* mRNA level is nearly 6-fold of that of *Rev-erbβ*, a closely related gene of *Rev-erbα* (Fig. 1B). Both *Rev-erbα* and *Rev-erbβ* are expressed at higher levels than the core clock gene *Bmal1* that is expressed at comparable level with the myogenic factor *Myod1*. The relative expression of *Rev-erbα* transcript to *Rev-erbβ* and *Bmal1* is similar in C2C12 myoblasts, although lower than that of the *Myod1* (Fig. 1C). To explore potential Rev-erbα involvement in myogenic response *in vivo*, we examined the dynamics of its expression following cardiotoxin (CTX)-induced muscle injury. CTX elicits regenerative myogenic repair, as indicated by strong myogenin induction at 5 and 8 days post injury, which gradually declines till day 15 (Fig. 1D). In sharp contrast to myogenin induction, Rev-erbα protein was markedly suppressed at day 5, with nearly abolished expression at 8 days after injury and recovered slightly at day 15. This finding suggests muscle damage or its associated myogenic signals may inhibit Rev-erbα. Indeed, treatment of primary myoblasts with crushed muscle extract (CME), obtained from lightly traumatized muscle that is known to contain myogenic stimuli^31^, results in significant down-regulation of *Rev-erbα* mRNA (Fig. 1E). The suppression of Rev-erbα occurs early at 6 hours after CME treatment and precedes the induction of myogenic markers at 12 hours, including *myogenin* and *embryonic myosin heavy chain* genes *(Myhc3*). Thus, Rev-erbα inhibition by muscle injury may occur as an early response to myogenic stimuli. One of the known mitogenic component of CME tested, Fgf2, can indeed suppress Rev-erbα protein at either a low or high concentration in primary myoblast cultures (Fig. 1F).

### Gene expression profiling reveals Rev-erbα regulation of Wnt and proliferative pathways

**Figure 2:**
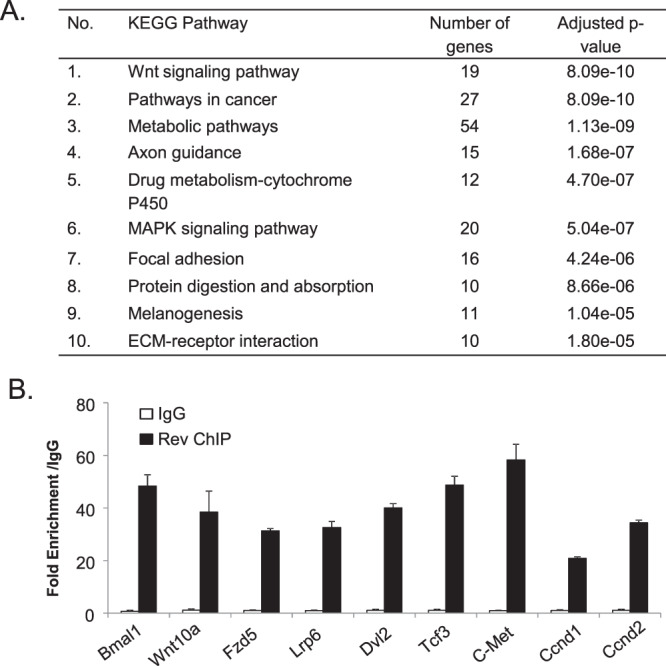
Rev-erbα transcriptional regulation of the Wnt signaling pathway and cell cycle regulators. (**A**) KEGG pathway gene enrichment analysis of differentially expressed genes in *Rev*^*−/−*^ vs. WT primary myoblasts. Genes that were significantly regulated with fold change ≥ 1.5 and adjusted P ≤ 0.05 from microarray analysis were analyzed (n = 3). Top 10 most significantly regulated biological pathways ranked by adjusted P values are listed. The dataset is deposited as NCBI GSE110765. (**B**) ChIP-qPCR analysis of Rev-erbα occupation of identified RORE sites in regulatory regions ±1 kb of candidate target genes in Wnt signaling or cell cycle regulators in proliferative C2C12 myoblasts (n = 3). Values are expressed as fold enrichment over IgG and known Rev-erbα binding site in Bmal1 first intron was included as positive control.

We performed gene expression profiling to explore *Rev-erbα* functions in MPC using proliferative satellite cell-derived primary myoblasts from *WT* and *Rev*^*−/−*^ mice. Differentially expressed genes, with fold change ≥ 1.5, were subjected to KEGG pathway analysis to identify *Rev-erbα-*differentially regulated biological functions. Top 15 ranked biological processes were listed (Fig. 2A), with the complete dataset deposited as NCBI GEO GSE110765. Consistent with the established metabolic functions of Rev-erbα, metabolic pathways are shown as top *Rev-erbα*-regulated processes in these cells, while potentially novel *Rev-erbα*-modulated functions, such as axon guidance, drug metabolism and protein metabolism, were identified. Notably, Wnt signaling and cancer pathways are ranked as the top two *Rev-erbα-*regulated pathways. As Wnt pathway is a major developmental signal that drives muscle development and repair^32–34^, Rev-erbα regulation of this cascade implicates its potential functions in myogenenic differentiation. In contrast, *Rev-erbα* differentially-regulated targets in cancer pathways are critical cell cycle regulators that could be involved in MPC proliferation. Additional pathways are identified that could also modulate myogenesis, including MAPK and actin cytoskeleton regulations^35,36^. Based on this analysis, we next examined potential Rev-erbα regulation of myogenesis through the Wnt and proliferative pathways.

Based on Rev-erbα differentially-regulated transcripts revealed by gene expression profiling, we next identified its candidate target genes in the Wnt and proliferative pathways. Following screening for putative consensus Rev-erbα binding sequence ROR-response element (RORE) within the +2 kb promoter region plus 1 kb downstream of transcription start site among these genes, ChIP-qPCR was performed to detect direct Rev-erbα chromatin occupancy on identified regulatory sites (Fig. 2B). Indeed, using the RORE Rev-erbα binding site on its known target Bmal1 first intron as a positive control, robust enrichment of chromatin association comparable to the known Bmal1 site were detected in a number of genes in Wnt signaling cascade as well as cell cycle regulators (c-Met, Cyclin D1 and D2). The striking finding of direct Rev-erbα transcriptional control of distinct steps along the Wnt pathway, including the ligand Wnt10a, receptors (Fzd5, Lrp6, Dvl2), and the transcription mediator (Tcf3), suggests that it may exert robust coordinated regulation of Wnt signaling, whereas its control of multiple proliferative genes indicates potential inhibitory effect on cell cycle.

### Ablation of Rev-erbα promotes myogenic gene expression in skeletal muscle and myoblast proliferation

**Figure 3:**
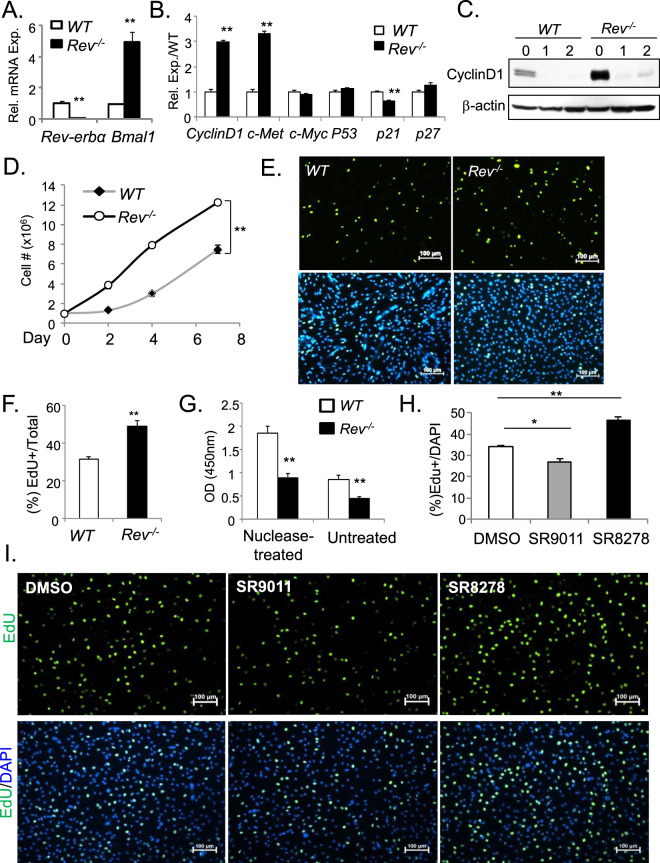
Rev-erbα inhibits myoblast proliferation. (**A**) RT-qPCR analysis of *Rev-erbα* and *Bmal1* mRNA in quadriceps of WT and *Rev*^*−/−*^ mice (n = 6/group). (**B**–**G**) Genetic loss-of-function of *Rev-erbα* promotes proliferation of primary myoblasts. (**B**) RT-qPCR analysis of expression of cell cycle regulators (n = 4). (**C**) Immunoblot analysis of Cyclin D1 protein level before and during differentiation in WT and *Rev*^*−/−*^ primary myoblasts (n = 3/time point). (**D**) Growth rate of primary myoblasts as assessed by increase in cell numbers monitored for 7 days in culture. Cells were seeded at a starting density of 1 × 10^6^/10 cm plate (n = 4/time point). **p ≤ 0.01 by one-way ANOVA. (**E**,**F**) Representative images of EdU labeling with corresponding DAPI image to assess proliferation (**E**) with quantification normalized to total cell numbers assessed by DAPI (F, n = 6). (**G**) Analysis of apoptosis by quantitative TUNEL assay in primary myoblasts (n = 5/group). **P < 0.01 *Rev*^*−/−*^ vs. WT by Student’s t test. (**H**,**I**) Effects of Rev-erbα ligands, agonist SR9011 and antagonist SR8278, on primary myoblast proliferation, as represented by quantification of percent of EdU+ cells/DAPI (H, n = 6/treatment group), with representative images of EdU labelling and DAPI shown (I, 10X). Cells were treated with DMSO (0.1%), SR9011 (10 nM) or SR8278 (10 nM) for 24 hours prior to EdU labelling for 6 hours. ***P ≤ 0.05 or 0.01 treated vs. DMSO by Student’s t test.

Global *Rev-erbα-null* (*Rev*^*−/−*^) mice were generated as previously described^23^. In *Rev*^*−/−*^ mutant mice, *Rev-erbα* transcript in representative mixed fiber type muscle quadriceps is undetectable (Fig. 3A). Due to the loss of Rev-erbα repression activity on its direct target gene, *Bmal1* mRNA level is markedly up-regulated ~5-fold as compared to the WT, as expected. The loss of *Rev-erbα* does not affect muscle development and growth, and histological analysis of quadriceps muscle cross sections of 8-week-old *Rev*^*−/−*^ mice did not reveal obvious defects.

As findings from the microarray study suggested, we further validated *Rev-erbα* transcriptional regulation of cancer pathway genes by RT-qPCR analysis. *Rev-erbα*-deficient myoblasts display ∼3-fold inductions of *Cyclin D1* and *c-Met*, whereas the cell cycle inhibitor *p21* was significantly suppressed (Fig. 3B). Furthermore, Cyclin D1 protein level in proliferating *Rev*^*−/−*^ myoblasts is markedly elevated as compared to WT prior to myogenic induction (D0 level prior or differentiation, Fig. 3C), although it can be similarly suppressed upon differentiation. We next tested whether *Rev-erbα* transcriptional control of cancer/proliferative pathways impacts proliferation, and found that primary myoblasts lacking *Rev-erbα* display an accelerated growth rate that nearly doubles that of the WT cells after seven days in culture (Fig. 3D). Direct examination of proliferative myoblasts by EdU incorporation revealed a significant 54.8% increase of proliferation rate in *Rev*^*−/−*^ *cells* as compared to WT controls (Fig. 3E,F). In addition, quantitative TUNEL assay to assess apoptotic rate in *Rev*^*−/−*^ cells these cells found a ~53% reduction under with basal untreated, or nuclease-treated conditions (Fig. 3G).

The genetic loss-of-function studies indicate that *Rev-erbα* negatively modulates myoblast proliferation. As the activity of Rev-erbα is amenable to modulation by synthetic ligands^21,22^, we tested whether pharmacological interventions of Rev-erbα affect myoblast proliferation, and found that activation of Rev-erbα by synthetic agonist SR9011 significantly attenuates, whereas its inhibition by an antagonist SR8278 augments primary myoblast proliferation (Fig. 3H,I). Thus, findings from genetic and pharmacological manipulations collectively demonstrate that *Rev-erbα* inhibits MPC proliferation.

### Rev-erbα suppresses Wnt pathway gene expression and signaling activity

**Figure 4:**
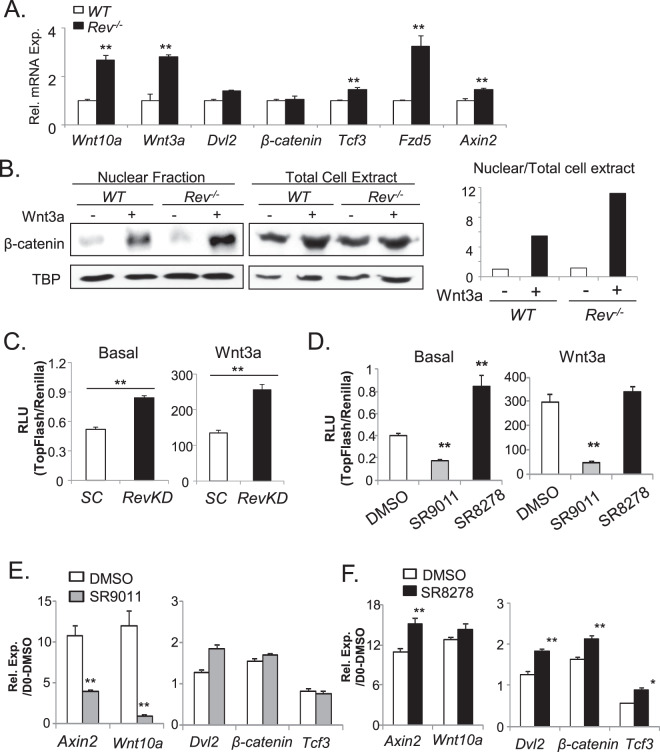
Rev-erbα regulation of Wnt pathway gene expression and signaling activity. (**A**) RT-qPCR analysis of Wnt pathway gene expression in WT and *Rev*^*−/−*^ primary myoblasts (n = 4). (**B**) Wnt signaling activity as assessed by immunoblot analysis of nuclear translocation of β-catenin in WT and *Rev*^*−/−*^ primary myoblasts. The ratio of nuclear to cytosolic β-catenin protein levels under basal and Wnt3a-stimulated conditions were calculated after normalization to TBP as loading control. (**C**) Wnt signaling activity was assessed by TopFlash-Luc reporter under basal and Wnt3a-stimulated conditions in C2C12 myoblasts with stably transfected scrambled control (SC) shRNA or Rev-erba knockdown (RevKD) shRNA (n = 5). **p ≤ 0.05 by Student’s t test. (**D**) The effects of Rev-erbα ligands, agonist SR9011 and antagonist SR8278, on Wnt signaling activity were assessed by TopFlash luciferase reporter assay in C2C12 myoblasts (n = 5/group). Ligands were added 8 hours prior to overnight Wnt3a (40%) stimulation before luciferase assay. **P < 0.01 vs. DMSO control by Student’s t test. (**E**,**F**) The effect of agonist SR9011 (**E**), and antagonist SR8278 (**F**) on Wnt signaling pathway gene expression as analyzed by RT-qPCR analysis, at 4 days of C2C12 myoblast differentiation (n = 3). ***P ≤ 0.05 or 0.01 vs. DMSO control.

As microarray study indicate that Wnt pathway is a top *Rev-erbα*-regulated process, we performed RT-qPCR analysis among differentially regulated genes identified and found that multiple components in the canonical Wnt cascade are significantly induced in *Rev*^*−/−*^ myoblasts as compared to WT (Fig. 4A). These genes include Wnt ligands, *Wnt10a* and *Wnt3a*, the Wnt receptor, *Fzd5*, and the Wnt signaling transcription mediator *Tcf4*. Of note, loss of *Rev-erbα* does not alter the transcript level of *Dishevelled 2* (*Dvl2)* and *β-catenin*. Expression of the Wnt target gene, *Axin2*, is also up-regulated, suggesting increased Wnt signaling activity in *Rev-erbα*-deficient myoblasts. Canonical Wnt signaling is transmitted through the inhibition of GSK-3β activity upon Wnt ligand stimulation and the subsequent nuclear translocation of β-catenin to activate TCF-induced transcription^37^. We thus determined Wnt signaling activity through analysis of β-catenin nuclear translocation in response to Wnt3a. As shown by immunoblot analysis of nuclear fraction vs. total cellular protein extract in WT cells (Fig. 4B), Wnt activity as assessed by nuclear β-catenin level is low in the absence of Wnt, and Wnt3a stimulation elicited a robust ~6-fold nuclear accumulation, as expected. In contrast, although the basal level of nuclear β-catenin remains low in *Rev*^*−/−*^ myoblasts, Wnt3a-stimulated nuclear translocation is markedly augmented, reaching ∼70% higher than that of the WT as shown by quantitative analysis. Using a Wnt-responsive TopFlash-Luciferase reporter to monitor TCF-mediated transcriptional activity^16^, we further examined whether Rev-erbα inhibition by stable shRNA knockdown (*RevKD*) affects Wnt signaling in C2C12 myoblasts, generated using clonal selection after transfection as previously^16^. As shown in Fig. 4C, Wnt signaling as detected by the reporter was significantly higher in *RevKD* cells, with Wnt3a-stimulated activity increased ~90% as compared to cells with scrambled control shRNA (SC).

Using this approach, we further determined the effects of pharmacological modulations of Rev-erbα activity on Wnt signaling. As shown by the Wnt-responsive luciferase reporter activity, Rev-erbα agonist, SR9011, markedly suppressed Wnt signaling at basal and Wnt3a-stimulated conditions (Fig. 4D). In contrast, the *Rev-erbα* antagonist, SR8278, augmented TopFlash reporter activity by 2-fold at basal state, although not under Wnt3a-stimulated state likely due to the saturated transcriptional activity levels induced by Wnt3a. In line with the observed effects of Rev-erbα ligands on Wnt signaling, SR9011 markedly inhibited the expression of Wnt pathway components, *Axin2* and *Wnt10* (Fig. 4E). In contrast, SR8278 was able to up-regulate the expression of *Axin2*, *Dvl2*, *β-catenin* and *Tcf3* genes within the Wnt cascade (Fig. 4F).

### Rev-erbα inhibits myogenic differentiation

**Figure 5:**
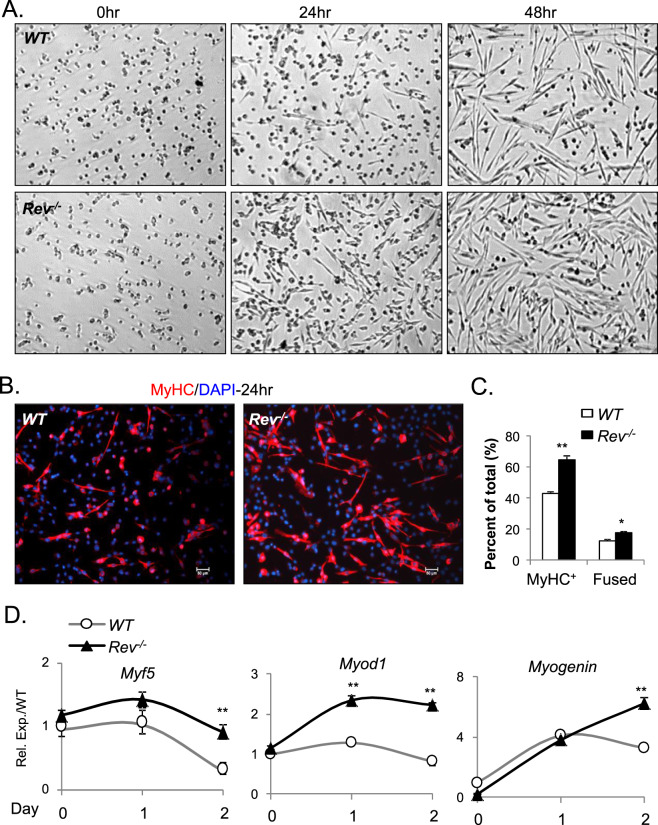
Loss of *Rev-erbα* enhances myogenic differentiation of *Rev*^*−/−*^ primary myoblasts. (**A**) Representative phase-contrast images of morphologic progression of WT and *Rev*^*−/−*^ primary myoblasts subjected to 2% horse serum-induced myogenic differentiation for 48 hours. (**B**,**C**) Representative images of MyHC immunostaining at 24 hours after myogenic induction (B, 10X). Quantification of total MyHC+ and fused myofibers (myofibers containing ≥ 2 nuclei) was shown in (**C**, 20X, n = 5). (**D**) RT-qPCR analysis of myogenic transcription factor induction during WT and *Rev*^*−/−*^ primary myoblasts differentiation over 2 days (n = 3).

As *Rev-erbα* inhibits Wnt signaling^32–34^, we postulate this regulation may lead to suppression of myogenic differentiation. We thus tested *Rev-erbα*-deficient primary myoblasts differentiation upon 2% horse serum induction, and found that the loss of *Rev-erbα* accelerated their morphological transformation toward organized myotubes (Fig. 5A). The advanced differentiation is further validated by a ~60% increase in the number of MyHC^+^ myofibers by immnostaining (64.4% *vs*. 42.8%) at 24 hours upon differentiation, together with higher percentage of fused myofibers containing multiple nuclei (Fig. 5B,C). Accompanying the accelerated morphological transformation, *Rev*^*−/−*^ myoblasts display elevated expression of *Myf5*, *Myod* and *myogenin* during the differentiation time course as compared to WT cells, particularly at late differentiation stage of day 2 (Fig. 5D).

**Figure 6:**
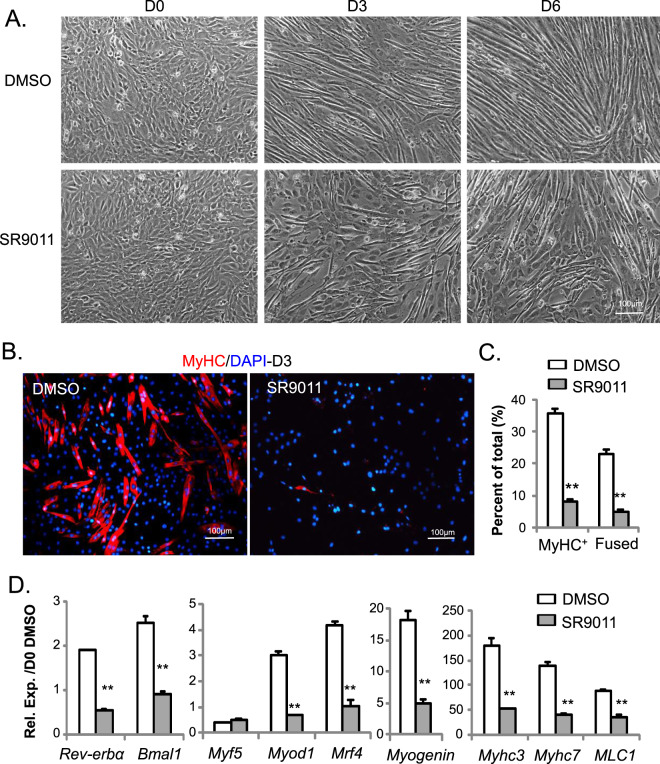
Activation of Rev-erbα by agonist SR9011 inhibits myogenic differentiation. (**A**–**C**) Inhibition of Rev-erbα agonist SR9011 on morphological progression of C2C12 myogenic differentiation for 6 days, as shown by representative phase-contrast images (**A**). Effect of SR9011 on myogenic differentiation as indicated by immunostaining of MyHC in day 3-differentiated C2C12 on chamber slides (**B**), with quantification of total MyHC+ and fused myofibers (**C**, n = 5/group) in the presence of vehicle (DMSO) or SR9011. DMSO (0.1%) or SR9011 (10 nM) were added 8 hours before myogenic induction and maintained throughout differentiation. (**D**) RT-qPCR analysis of SR9011 effect on suppressing myogenic gene expression at day 4 of C2C12 differentiation (n = 3). ***P ≤ 0.05 or 0.01 SR9011 vs. DMSO by Student’s t test.

We next examined whether *Rev-erbα* ligands affects myoblast differentiation. Opposite to the effect of genetic loss-of-function, activation of Rev-erbα by SR9011 markedly suppresses the appearance of myotubes at day 3 of early myoblast differentiation in C2C12 cell line, as compared to the DMSO-treated controls (Fig. 6A). At 6 days post myogenic induction when myotube formation of DMSO-treated myoblasts is largely complete, there is still a significant percentage of cells in SR9011-treated culture remain undifferentiated. Immunostaining of MyHC at 3 days post-differentiation confirmed an evident defect in myotube formation, as SR9011 nearly abolished the mature myocyte marker expression (Fig. 6B,C). Gene expression analysis revealed that SR9011 repressed the expression of known Rev-erbα target genes, *Rev-erbα* itself and *Bmal1*, indicating the activation of Rev-erbα transcription activity. Furthermore, myogenic regulatory factors (*Myod1, Mrf4, myogenin*) and myocyte-specific markers (*Myhc3*, *Myhc7* and *MLC1*) were markedly down-regulated by SR9011 as compared to control (Fig. 6D), corroborating the impaired differentiation phenotype in SR9011-treated cells.

**Figure 7:**
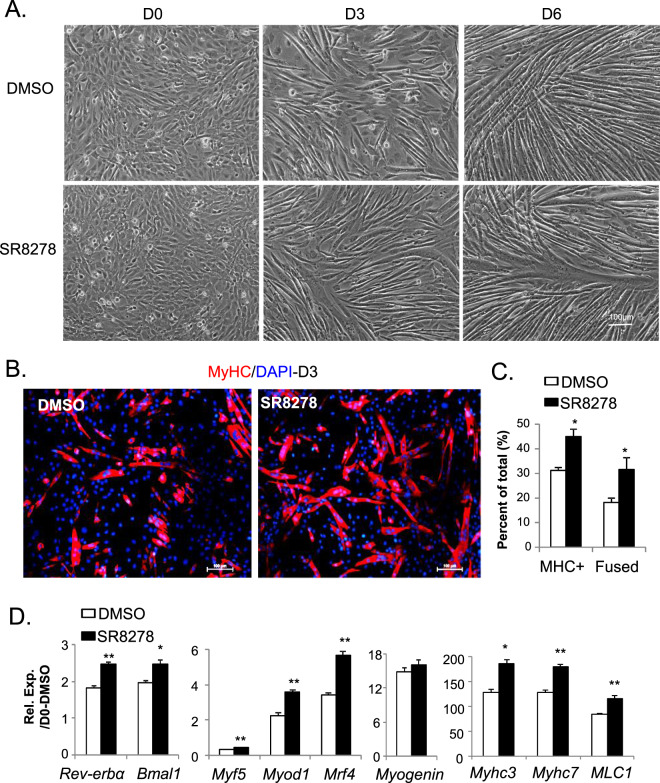
Inhibition of Rev-erbα by antagonist SR8278 promotes myogenic differentiation. (**A**–**C**) Advanced morphological progression of C2C12 myoblast differentiation treated by SR8278 as compared to DMSO, as shown by representative phase-contrast image at day 0 to 6 in culture (**A**). Effect of SR8278 on myogenic differentiation as indicated by MyHC immunostaining in day 3-differentiated C2C12 on chamber slides (**B**), with quantification of total MyHC+ and fused myofibers (**C**, n = 5/group), in the presence of vehicle (DMSO) or SR8278. Cells were treated with DMSO (0.1%) or agonist SR8278 (10 nM) 8 hours prior to induction of differentiation and maintained throughout differentiation. (**D**) RT-qPCR analysis of effect of SR8278 on myogenic gene expression at day 4 of C2C12 differentiation (n = 3). ***P ≤ 0.05 or 0.01 SR8278 vs. DMSO by Student’s t test.

In contrast to SR9011, the Rev-erbα antagonist, SR8278, promotes the morphological changes toward mature myotube formation in C2C12 myoblasts relative to DMSO-treated controls (Fig. 7A). SR8278 treatment significantly increased the percentage of MyHC^+^ myofibers and fused myofiber containing multiple nuclei upon 3 days of myogenic induction (Fig. 7B,C). The accelerated morphological differentiation was accompanied by augmented expression of myogenic regulatory factors (*Myf5, Myod1 and Mrf4*) and myocyte-specific markers (*Myhc3, Myhc7 and MLC1*, Fig. 7D). SR8278 effectively blunted the transcription repressor activity of Rev-erbα, as indicated by the induction of *Rev-erbα* and *Bmal1* transcripts. Thus, both genetic and pharmacological findings demonstrate that *Rev-erbα* inhibits MPC differentiation.

### Loss of Rev-erbα augments muscle regeneration and satellite cell proliferative expansion

**Figure 8:**
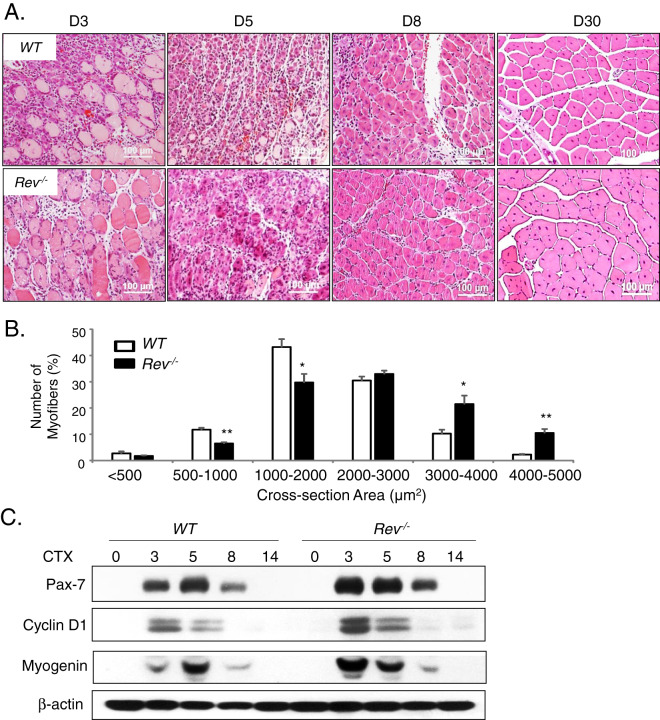
Loss of Rev-erbα enhances muscle regeneration. (**A**) Representative images of H/E histological analysis of muscle regeneration at 3, 5, 8 and 30 days. The number of mice used for these analysis are: day 0 (WT n = 6, *Rev*^*−/−*^ n = 6), day 3 (WT n = 6, *Rev*^*−/−*^ n = 5), day 5 (WT n = 7, *Rev*^*−/−*^ n = 6), day 8 (WT n = 5, *Rev*^*−/−*^ n = 6), day 14 (WT n = 6, *Rev*^*−/−*^ n = 5), day 30 (WT n = 6, *Rev*^*−/−*^ n = 8). (**B**) Quantitative analysis of regenerated myofiber diameter distribution at 30 days after cardiotoxin injury in WT (n = 6) and *Rev*^*−/−*^ mice (n = 8). Values are represented as the percentage of number of myofibers within the indicated range over total number of myofibers. Three representative 10X sections with ~450 total myofibers were counted for each mouse in the group. Non-parametric Kruskal–Wallis ANOVA for dataset p-value < 0.0001, and non-parametric Mann-Whitney test are indicated for individual cross section area category: ***P ≤ 0.05 or 0.01 *Rev*^*−/−*^ vs. WT. (**C**) Immunoblot analysis of protein levels of cell cycle regulator and myogenic marker in regenerating TA muscle lysate at indicated time points after CTX injury. Protein samples were pooled from each group of WT or *Rev*^*−/−*^ at indicated time point as shown in (**A**).

**Figure 9:**
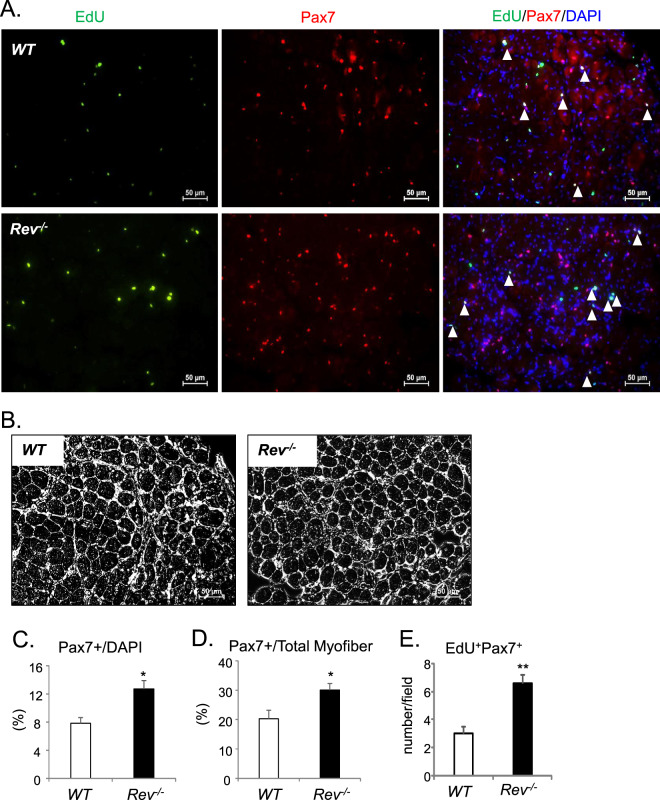
Loss of Rev-erbα promotes satellite cell proliferative expansion during muscle regeneration. (**A**,**B**) Representative images of Pax7 immunofluorescent staining to identify total and EdU staining to label proliferative satellite cells, in day 8-regenerating TA post CTX injury (**A**). Arrowheads denote co-localization of Pax7 (red), EdU (green) with DAPI (blue) that identifies proliferating satellite cells. Phase contrast images of corresponding fields were shown in (**B**). (**C**–**E**) Quantification of number of Pax7+ satellite cells expressed as percentage of total nuclei (**C**), or normalized as the percentage of total number of myofibers (**D**); and the number of proliferative satellite cells as indicated by co-localized Pax7+ and EdU+ staining (**E**, n = 4). ***P ≤ 0.05 or 0.01 *Rev*^*−/−*^ vs. WT by Student’s t test.

Upon muscle injury, the orderly activation, proliferation, and differentiation of MPCs, satellite cells and its progenies, critically determine the regenerative repair response^15,38^. Based on our findings of *Rev-erbα* inhibition of proliferation and differentiation in myoblasts, loss of these activities may promote muscle regeneration *in vivo*. We thus test the regenerative response of *WT* and *Rev*^*−/−*^ animals to cardiotoxin (CTX)-induced muscle injury CTX was injected throughout the length and thickness of Tibialis Anterior muscle to ensure uniform injury response, as described^17^, and muscle regenerative repair was monitored for 30 days after injury. Initial histological examination of the regeneration time course suggests enhanced repair in *Rev-erbα*-null mutants as compared to WT (Fig. 8A). At the peak of new myofiber synthesis at day 5, when nascent myofibers of WT are small, new *Rev*^*−/−*^ myofibers appear larger with robust basophilic staining. More advanced maturation of well-formed new myofibers containing central nuclei is evident by day 8 post-injury. At day 30 when the normal morphology of the muscle is largely restored by regenerated myofibers, quantitative analysis of cross-section areas reveal significantly skewed distribution toward larger myofibers in *Rev*^*−/−*^ mice as compared to the WT (Fig. 8B). The expression kinetics of key factors involved in MPC proliferative and myogenic response during the regeneration time course was examined, as shown in Fig. 7C. Upon muscle damage, satellite cells are activated to proliferate and expand to provide new myonuclei for regenerative repair. This normal response of satellite cell expansion in WT mice is reflected by the strong induction of the satellite cell marker, Pax7 protein, which peaks at 5 days after injury, gradually declines at day 8 and terminates at 14 days (Fig. 8C). In comparison, *Rev-erbα*-null mice display elevated Pax7 induction throughout the satellite activation stages at day 3 and 5, with its peak level advanced to day 3. The continued high expression of Pax7 in *Rev-erbα*-null mice as compared to WT at day 8 post-injury indicates a potentially more sustained satellite cell proliferative expansion phase. In line the expression kinetics of Pax7, Cyclin D1 induction at day 3 and 5 are markedly higher in *Rev*^*−/−*^ mice, suggesting increased proliferative activities of cycling myogenic progenitors. Interestingly, myogenin levels in these mice exhibit a similarly augmented kinetics with an early peak expression at day 3, suggesting concomitantly enhanced myogenic maturation of activated progenitors. As the elevated Pax7 protein observed at day 8 near the end of the proliferative expansion suggests more abundant Pax7-expressing satellite cells, we determined satellite cell numbers directly through Pax7 immunofluorescence staining in muscle sections (Fig. 9A). Consistent with the low expression of Pax7 in WT mice at day 8 post injury, the number of Pax7+ satellite cells detected is limited. In contrast, ~50% higher amount of satellite cells was found in regenerating *Rev*^*−/−*^ muscle than the WT (Fig. 9B,C). Furthermore, identification of proliferating satellite cell by EdU incorporation *in vivo* revealed nearly doubling of the number of cells with EdU^+^/Pax7^+^ co-staining in *Rev*^*−/−*^ muscle as compared to WT (Fig. 9D). These findings demonstrate that, in line with the observed functions of *Rev-erbα* in suppressing MPC proliferative and myogenic activities, the loss of *Rev-erbα* augments satellite cell-mediated regenerative repair *in vivo*.

## Discussion

Maintenance of proper MPC proliferation and differentiation functions critically determines skeletal muscle growth and repair^15,38^. Our study identifies that the clock repressor *Rev-erbα* is an inhibitory factor of MPC proliferative and myogenic properties, and these functions suppress skeletal muscle regeneration *in vivo*.

The appropriate balance and separation between proliferation and differentiation of MPCs ensures the expansion of the myogenic precursor pool and their subsequent maturation for muscle repair. Manipulations that promote MPC proliferation alone hinder myogenic differentiation, whereas enhancing differentiation limits MPC proliferative growth^39–42^. Our current study uncovers an intriguing finding that the loss of *Rev-erbα* function coordinately facilitates MPC proliferation with myogenic progression. Similar to the known effects of IGF-1 on myoblasts^43–45^ but distinct from typical cell cycle regulators or myogenic factors, these actions likely contributed to the enhanced myogenic repair observed in *Rev-erbα-null* mice in concert. These inhibitory Rev-erbα functions in regenerative myogenesis could be a mechanism to prevent excessive tissue remodeling in response to injury and maintain tissue homeostatic control. On the other hand, suppressing Rev-erbα activity may have the potential to promote skeletal muscle regenerative capacity. Given that we have demonstrated previously that *Bmal1* promotes MPC myogenic activities^16,17^, *Rev-erbα* may function in concert with *Bmal1* or additional clock regulators in MPC to orchestrate skeletal muscle regenerative myogenesis.

Through gene expression profiling, we uncovered a novel mechanistic link between Rev-erbα and the Wnt signaling pathway. We identified that genes along distinct steps of the Wnt cascade are direct Rev-erbα target genes with RORE consensus binding sites. This raises an intriguing possibility whether RORα, the transcription activator of the RORE elements and critical for the core clock loop^1^, may exert positive transcriptional control of Wnt and proliferative pathway to modulate myogenesis. Future studies of potential RORα functions in myogenic pathways would lend additional support to circadian clock mechanisms involved. And, as another ligand-modulated nuclear receptor, RORα regulations of myogenic repair may also reveal novel therapeutic targets for muscle diseases^46–48^. Interestingly in previous studies, we found that *Bmal1* exerts positive transcriptional control of key signaling nodes of the Wnt pathway to promote myogenesis^16^. The new findings in our study suggest that it is possible that the *Bmal1* activation together with *Rev-erbα* repression, likely at distinct times in a circadian cycle, may underlie rhythmic expression of Wnt pathway genes, as we demonstrated in synchronized myoblasts in the previous study^16^. As Wnt pathway is a major developmental signal that drives myogenesis, its modulation by clock regulators may confer an additional layer of control to fine-tune the myogenic cascade. A study by Downes *et al*. found that ectopic overexpression of a *Rev-erbα* dominant negative mutant promotes myogenic differentiation^49^, a finding that is consistent with our current results. Notably in our study, the effect of loss of endogenous Rev-erbα on muscle regeneration *in vivo* is not as marked as demonstrated by the dominant negative mutant. It is possible that a closely related Rev-erbβ gene may have partially overlapping myogenic functions with Rev-erbα that provides functional redundancy in muscle regeneration^18^.

An intimate link between circadian clock and cell cycle regulation has long been established^50,51^. CLOCK-controlled mRNA in growth and differentiation comprise ~15% of all transcripts in skeletal muscle^7^. Studies of *Bmal1* on MPC proliferation and satellite cell expansion^16,17^ are in line with this notion^11,12^. Based on the significant overlap of Rev-erbα and Bmal1 genomic targets and their opposing functions in the clock circuit^18^, Rev-erbα may inhibit MPC proliferation. Although few studies directly examined Rev-erbα function in proliferative pathways, several observations to date supports its involvement. *Rev-erbα*-deficiency increases hippocampal neuronal progenitor proliferation^52^, whereas its activation by SR9011 inhibits breast cancer cell proliferative growth^53^. With our finding of Rev-erbα inhibitory effect on myoblast proliferation, it may modulate proliferative behaviors in a broad array of tissue/cell types. Many of Rev-erbα differentially-regulated genes in cancer/proliferative pathway we identified shares significant overlap with a published ChIP-Seq dataset^18^. Indeed, our analysis of chromatin association of Rev-erbα among its differentially-regulated genes indicate that several cell cycle regulators are its direct transcriptional targets in proliferative myoblasts, although additional targets of Rev-erbα in proliferative pathway may be uncovered through global ChIP-Seq approaches in future studies.

*Rev-erbα* expression was strongly suppressed by muscle injury stimuli. This finding suggests that physiological inhibitory effects of Rev-erbα on MPC proliferation and myogenesis may help maintain a quiescent state, and that its relief upon injury facilitates myogenic activation in repair. Possibly due to its dynamic regulation by injury stimuli, no significant effect of *Rev-erbα* ablation on muscle growth was evident under normal condition. Potential functional redundancy between *Rev-erbα* and *Rev-erbβ* with overlapping genomic targets is another possibility. Current findings of Rev-erbα regulation of MPC behavior establish a basis for future in-depth analysis of molecular clock function in muscle stem cells. A growing body of evidence indicates that the circadian clock is required for coordinating stem cell response to external stimuli in tissue growth and remodeling processes^54–57^. Rev-erbα regulation of proliferation and differentiation pathways, as we uncovered in this study, may apply to a myriad of stem cell paradigms beyond skeletal muscle repair.

Rev-erbα inhibition of MPC behavior in isolated primary myoblasts suggests the cell-autonomous roles of Rev-erbα. Enhanced satellite cell proliferation in regenerative expansion we observed in Rev-erbα-null mice is consistent with this notion. Nonetheless, although MPC is major cellular source for muscle regeneration, optimal repair requires additional stem cell populations and other cell types including macrophages, fibroblasts, endothelial cells^38,58–61^. The global ablation of the Rev-erbα-null in our study therefore cannot exclude its involvement in additional processes of regeneration. Particularly, the known immune-modulatory activities of Rev-erbα may contribute to regenerative repair^62,63^. Future cell type-selective gene targeting studies are required to dissect the cell-autonomous Rev-erbα functions in MPC vs. other cell types in skeletal muscle repair.

As a “druggable” nuclear receptor, Rev-erbα activity is modulated by synthetic ligands^21,22^. Our *in vitro* studies of *Rev-erbα* antagonist SR8278 in myoblasts provide the evidence and mechanisms by which that pharmacological inhibition of *Rev-erbα* augments MPC myogenic activities. Consistent with our current findings, a recent study by Welch *et al*.^64^ tested SR8278 in mdx mice that resulted in functional improvement of dystrophic pathpophysiology. Thus, as these studies uncovered, there are potential therapeutic utilities of Rev-erbα antagonism in degenerative or dystrophic muscle diseases through stimulation of myogenic growth and repair mechanisms. Ultimately, better mechanistic understandings of Rev-erbα function in myogenic progenitor behavior may lead to the discovery of new strategies to enhance regenerative capacity for the maintenance of muscle mass.

## Supplementary information

Suppl Info
