# Author Correction: The Nuclear Receptor and Clock Repressor Rev-erbα Suppresses Myogenesis

**DOI:** 10.1038/s41598-022-08153-9

**Published:** 2022-03-22

**Authors:** Somik Chatterjee, Hongshan Yin, Weini Li, Jeongkyung Lee, Vijay K. Yechoor, Ke Ma

**Affiliations:** 1 Center for Diabetes Research, Department of Medicine, Houston Methodist Research Institute, Houston, TX 77030 USA; 2 Department of Cardiovascular Medicine, Third Affiliated Hospital, Hebei Medical University, Shijiazhuang, 050051 Hebei China; 3 Department of Diabetes Complications & Metabolism, Beckman Research Institute of City of Hope, Duarte, CA 91010 USA; 4 Diabetes and Beta Cell Biology Center, Division of Endocrinology, Diabetes & Metabolism, Department of Medicine, University of Pittsburgh, Pittsburgh, PA 15213 USA

Correction to: *Scientific Reports* 10.1038/s41598-019-41059-7, published online 14 March 2019

The original Article contained an error in Figure 8A, where D5 WT was incorrectly duplicated as Rev-/- D3. The original Figure 8 appears below.

**Figure 8:**
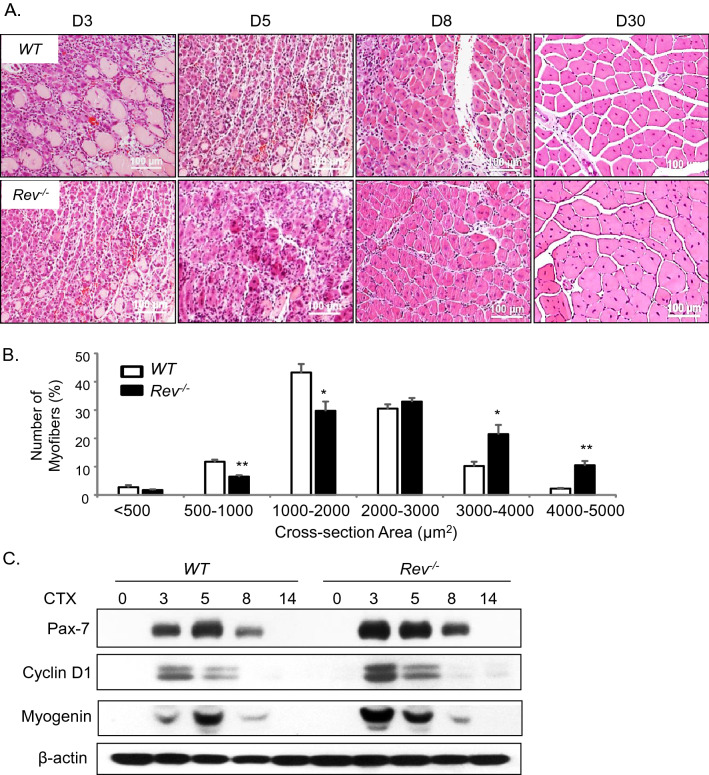
Loss of Rev-erbα enhances muscle regeneration. (**A**) Representative images of H/E histological analysis of muscle regeneration at 3, 5, 8 and 30 days. The number of mice used for these analysis are: day 0 (WT n = 6, Rev−/− n  = 6), day 3 (WT n = 6, Rev−/− n = 5), day 5 (WT n = 7, Rev−/− n = 6), day 8 (WT n = 5, Rev−/− n = 6), day 14 (WT n = 6, Rev−/− n = 5), day 30 (WT n = 6, Rev−/− n = 8). (**B**) Quantitative analysis of regenerated myofiber diameter distribution at 30 days after cardiotoxin injury in WT (n = 6) and Rev−/− mice (n = 8). Values are represented as the percentage of number of myofibers within the indicated range over total number of myofibers. Three representative 10X sections with ~ 450 total myofibers were counted for each mouse in the group. Non-parametric Kruskal–Wallis ANOVA for dataset p-value < 0.0001, and non-parametric Mann–Whitney test are indicated for individual cross section area category: ***P ≤ 0.05 or 0.01 Rev−/− vs. WT. (**C**) Immunoblot analysis of protein levels of cell cycle regulator and myogenic marker in regenerating TA muscle lysate at indicated time points after CTX injury. Protein samples were pooled from each group of WT or Rev−/− at indicated time point as shown in (**A**).

The original Article has been corrected.

