# Supplementary material for: The Nuclear Receptor and Clock Repressor Rev-erbα Suppresses Myogenesis
Source: Sci Rep. 2019 Mar 14;9:4585. doi: 10.1038/s41598-019-41059-7 (PMC6418265; doi:10.1038/s41598-019-41059-7)

The Nuclear Receptor and Clock Repressor Rev-erbα Suppresses Myogenesis

Somik Chatterjee[1,] Hongshan Yin[1,2], Weini Li[3], Jeongkyung Lee[4], Vijay K. Yechoor[4], Ke Ma[3#]

[1]Center for Diabetes Research, Department of Medicine, Houston Methodist Research Institute, Houston, TX, 77030

[2]Department of Cardiovascular Medicine, Third Affiliated Hospital, Hebei Medical University, Shijiazhuang, 050051, Hebei, China.

[3]Department of Diabetes Complications & Metabolism, Beckman Research Institute of City of Hope, Duarte, CA 91010

[4]Diabetes and Beta Cell Biology Center, Division of Endocrinology, Diabetes & Metabolism, Department of Medicine, University of Pittsburgh, Pittsburgh, PA

[#]: To whom correspondence should be addressed:

E-mail: ke.ma@coh.org

Phone: (626) 218-3796

Fax: (626) 218-4112

Supplemental Table 1. Primary antibodies list.

Antibody	Source	Cat#	Dilution
Rev-erba	Thermo Scientific	PA5-29865	1:1000
Pax7	Developmental studies Hybridoma Bank	Pax7	1:100
Cyclin D1 (92G2)	Cell Signaling	92G2	1:1000
Beta-catenin	BD Biosciences	610153	1:1000
GAPDH clone 6C5	Millipore	MAB374	1:1000
Myogenin	Millipore	MAB3876	1:200
Beta-actin	Abcam	AB3280	1:4000
TBP	Santa Cruz	SC-204	1:500

Supplemental Table 2. Primer sequence for qPCR analysis.

Genes		Sequences
Bmal1	Forward	CGCTTTCTGGAGGGTGTCCGC
	Reverse	TGCCAGGACGCGCTTGTACC
Wnt5a	Forward	TGGCATCCGGTGCACTGCAG
	Reverse	CCCTCCAGAAGGGTAGCACGCT
Fzd5	Forward	ACTCACCGTGCTAAGGATGGCTGT
	Reverse	TCACGCCTTTCATAACACATTCC
Wnt10α	Forward	CAACGCGTGCGCTCTGGGTA
	Reverse	TGGCTCAAGCCCTTTCCGCG
Dvl2	Forward	TCAGTTTGCGGGTGTGCGCAG
	Reverse	TTCGTCTCGCCTACACCACCG
Fzd5	Forward	AATCATGCAGGGGGCCCCGAA
	Reverse	CGACAAGCTAGGTACCTGTGGCG
TCF3	Forward	AGGGCCTGCCAGGGACATCA
	Reverse	GGATGGCCTCGTCCAAGCGG
β-catenin	Forward	CGCTTGGCTGAACCATCAC
	Reverse	GTTCCGCGTCATCCTGATAGT
Myod1	Forward	TGGCATCCGGTGCACTGCAG
	Reverse	CCCTCCAGAAGGGTAGCACGCT
Myf5	Forward	AGCTGCTGAGGGAACAGGTGGA
	Reverse	ATTCAGGCATGCCGTCAGAGCA
Axin2	Forward	TGACTCTCCTTCCAGATCCCA
	Reverse	TGCCCACACTAGGCTGACA
Myogenin	Forward	GTCCCAACCCAGGAGATCATT
	Reverse	AGTTGGGCATGGTTTCGTCT
MHC3	Forward	AAGGCCATCACTGACGCCGC
	Reverse	CGCCAGCTGCTCAGCCTCAT
MLC1	Forward	GCAACAGGAGGACTTCAAGGAGGC
	Reverse	ATTGGTGCCCAGAGCCCGGA
CyclinD1	Forward	GCGTACCCTGACACCAATCTC
	Reverse	CTCCTCTTCGCACTTCTGCTC
C-Met	Forward	TGGGCCGGCTTAACCAAGTGC
	Reverse	AGCACCACCTGCATGAAGCGA
P21	Forward	CCTGGTGATGTCCGACCTG
	Reverse	CCATGAGCGCATCGCAATC
c-Myc	Forward	CAACGACAGCAGCTCGCCCA
	Reverse	ACTAGGGGCTCAGGGCTGGC
P27	Forward	TCAAACGTGAGAGTGTCTAACG
	Reverse	CCGGGCCGAAGAGATTTCTG

Supplemental Table 3. Primer sequence for Rev-erbα ChIP-qPCR analysis.

Genes		Sequences
36B4	Forward	
	Reverse	
Rev-erbα	Forward	TGGCATCCGGTGCACTGCAG
	Reverse	CCCTCCAGAAGGGTAGCACGCT
Bmal1	Forward	ACTCACCGTGCTAAGGATGGCTGT
	Reverse	TCACGCCTTTCATAACACATTCC
Wnt10α	Forward	CAACGCGTGCGCTCTGGGTA
	Reverse	TGGCTCAAGCCCTTTCCGCG
Dvl2	Forward	TCAGTTTGCGGGTGTGCGCAG
	Reverse	TTCGTCTCGCCTACACCACCG
Fzd5	Forward	AATCATGCAGGGGGCCCCGAA
	Reverse	CGACAAGCTAGGTACCTGTGGCG
TCF3	Forward	AGGGCCTGCCAGGGACATCA
	Reverse	GGATGGCCTCGTCCAAGCGG
β-catenin	Forward	CGCTTGGCTGAACCATCAC
	Reverse	GTTCCGCGTCATCCTGATAGT
Myod1	Forward	TGGCATCCGGTGCACTGCAG
	Reverse	CCCTCCAGAAGGGTAGCACGCT
Myf5	Forward	AGCTGCTGAGGGAACAGGTGGA
	Reverse	ATTCAGGCATGCCGTCAGAGCA
Axin2	Forward	TGACTCTCCTTCCAGATCCCA
	Reverse	TGCCCACACTAGGCTGACA
Myogenin	Forward	GTCCCAACCCAGGAGATCATT
	Reverse	AGTTGGGCATGGTTTCGTCT
MHC3	Forward	AAGGCCATCACTGACGCCGC
	Reverse	CGCCAGCTGCTCAGCCTCAT
MLC1	Forward	GCAACAGGAGGACTTCAAGGAGGC
	Reverse	ATTGGTGCCCAGAGCCCGGA
CyclinD1	Forward	GCGTACCCTGACACCAATCTC
	Reverse	CTCCTCTTCGCACTTCTGCTC
C-Met	Forward	TGGGCCGGCTTAACCAAGTGC
	Reverse	AGCACCACCTGCATGAAGCGA
P21	Forward	CCTGGTGATGTCCGACCTG
	Reverse	CCATGAGCGCATCGCAATC
c-Myc	Forward	CAACGACAGCAGCTCGCCCA
	Reverse	ACTAGGGGCTCAGGGCTGGC
P27	Forward	TCAAACGTGAGAGTGTCTAACG
	Reverse	CCGGGCCGAAGAGATTTCTG

Original Western Data Scan

Fig. 1A Rev-erba

9AM 5PM 9AM 5PM 9AM 5PM 9AM 5PM 9AM 5PM 9AM 5PM 9AM 5PM

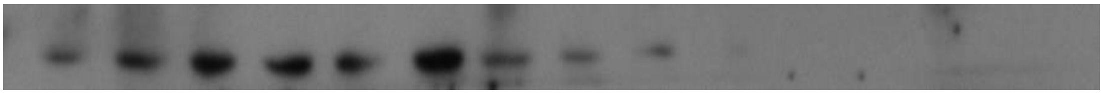

Fig. 1A GAPDH

9AM 5PM 9AM 5PM 9AM 5PM 9AM 5PM 9AM 5PM 9AM 5PM 9AM 5PM

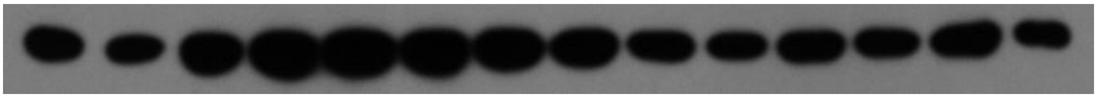

Fig. 2C CyclinD1

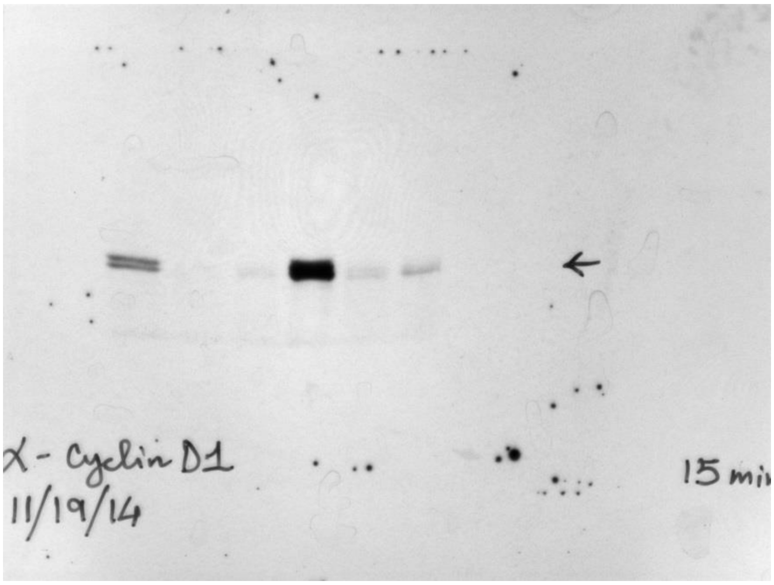

Fig. 2C b-Actin

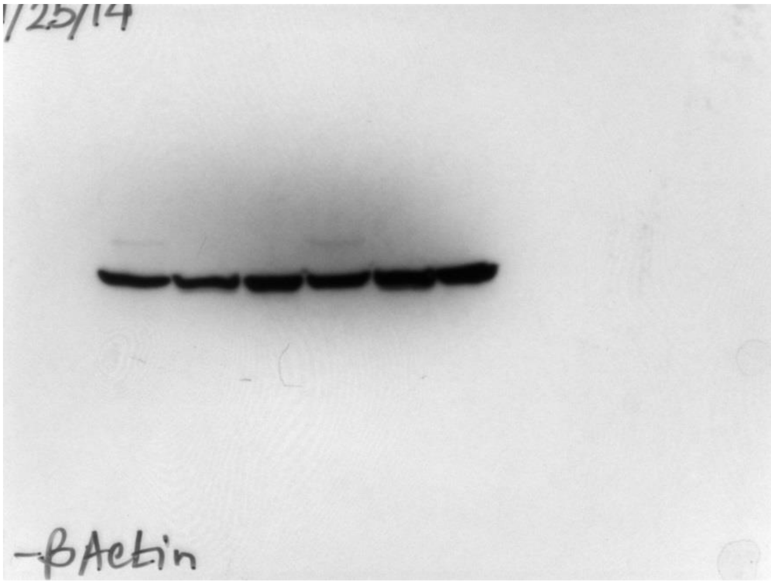

Fig. 3B b-Catenin

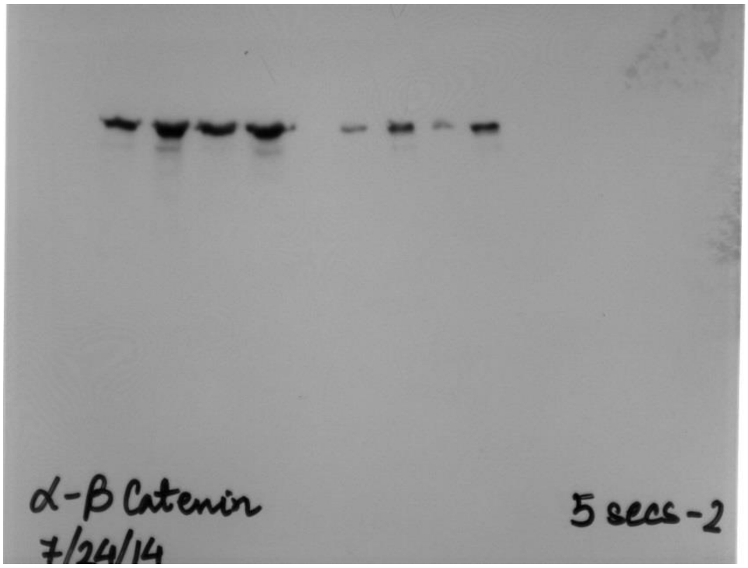

Fig. 3B TBP

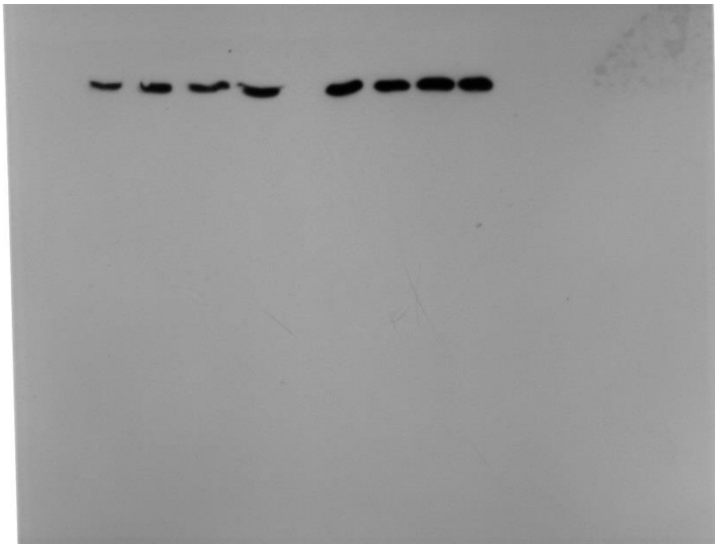

Fig. 7C Myogenin

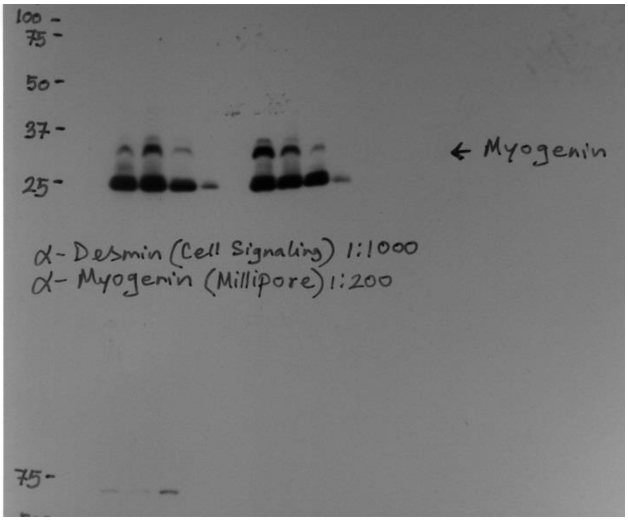

Fig. 7C CyclinD1

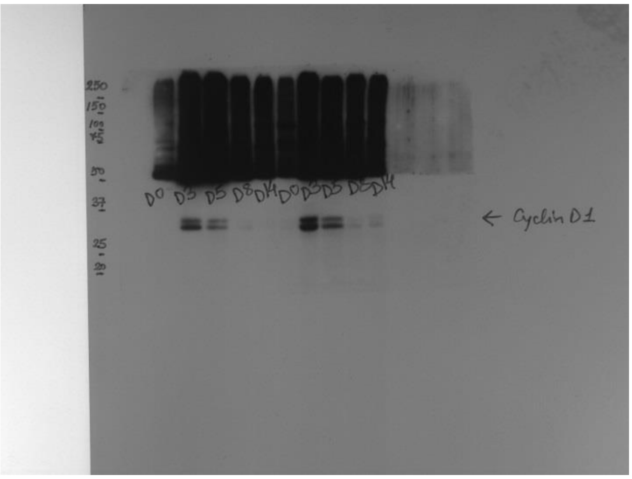

Fig. 7C Pax7 & b-actin

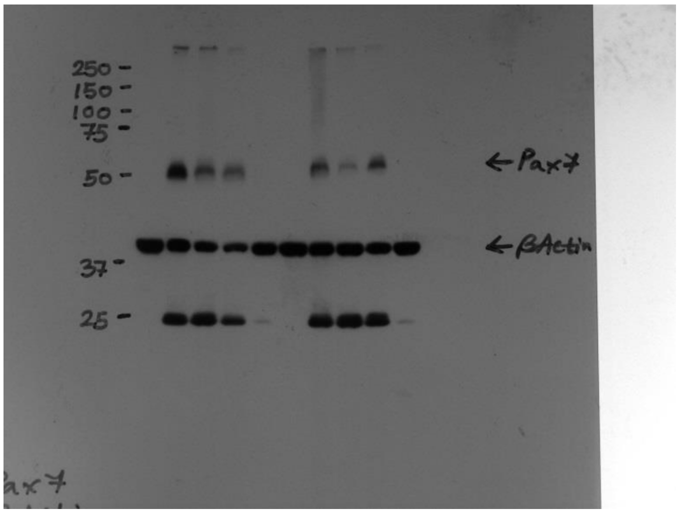

Supplement: Supplementary file 1 — Suppl Info [file 41598_2019_41059_MOESM1_ESM.pdf]
